# Predictive Models for Neonatal Follow-Up Serum Bilirubin: Model Development and Validation

**DOI:** 10.2196/21222

**Published:** 2020-10-29

**Authors:** Joseph H Chou

**Affiliations:** 1 Massachusetts General Hospital Boston, MA United States

**Keywords:** infant, newborn, neonatology, jaundice, neonatal, hyperbilirubinemia, neonatal, machine learning, supervised machine learning, data science, medical informatics, decision support techniques, models, statistical, predictive models

## Abstract

**Background:**

Hyperbilirubinemia affects many newborn infants and, if not treated appropriately, can lead to irreversible brain injury.

**Objective:**

This study aims to develop predictive models of follow-up total serum bilirubin measurement and to compare their accuracy with that of clinician predictions.

**Methods:**

Subjects were patients born between June 2015 and June 2019 at 4 hospitals in Massachusetts. The prediction target was a follow-up total serum bilirubin measurement obtained <72 hours after a previous measurement. Birth before versus after February 2019 was used to generate a training set (27,428 target measurements) and a held-out test set (3320 measurements), respectively. Multiple supervised learning models were trained. To further assess model performance, predictions on the held-out test set were also compared with corresponding predictions from clinicians.

**Results:**

The best predictive accuracy on the held-out test set was obtained with the multilayer perceptron (ie, neural network, mean absolute error [MAE] 1.05 mg/dL) and Xgboost (MAE 1.04 mg/dL) models. A limited number of predictors were sufficient for constructing models with the best performance and avoiding overfitting: current bilirubin measurement, last rate of rise, proportion of time under phototherapy, time to next measurement, gestational age at birth, current age, and fractional weight change from birth. Clinicians made a total of 210 prospective predictions. The neural network model accuracy on this subset of predictions had an MAE of 1.06 mg/dL compared with clinician predictions with an MAE of 1.38 mg/dL (*P*<.0001). In babies born at 35 weeks of gestation or later, this approach was also applied to predict the binary outcome of subsequently exceeding consensus guidelines for phototherapy initiation and achieved an area under the receiver operator characteristic curve of 0.94 (95% CI 0.91 to 0.97).

**Conclusions:**

This study developed predictive models for neonatal follow-up total serum bilirubin measurements that outperform clinicians. This may be the first report of models that predict specific bilirubin values, are not limited to near-term patients without risk factors, and take into account the effect of phototherapy.

## Introduction

### Neonatal Jaundice: Bilirubin Production and Clearance

Management of jaundice is one of the most common, yet vexing, problems in newborn medicine and requires consideration of the myriad contributors to the production and clearance of bilirubin [1]. If not recognized and managed appropriately, hyperbilirubinemia can result in permanent harm. A large proportion of neonatal readmissions is related to jaundice [2]. Bilirubin arises from the catabolism of iron protoporphyrin (heme) from hemoglobin in red blood cells. Unconjugated bilirubin is poorly water soluble and largely bound to albumin but is conjugated in the liver into a more water-soluble form more readily excreted in the bile and urine.

A number of physiological mechanisms put newborn infants at particular risk of developing jaundice in the first few days after birth, including increased red blood cell volume, higher red blood cell turnover, decreased hepatic uptake and conjugation of bilirubin, and increased enterohepatic circulation (intestinal hydrolysis of conjugated bilirubin resulting in reabsorption of unconjugated bilirubin). This initial imbalance of increased bilirubin production and decreased conjugation and clearance results in >80% of newborn infants born near or at term developing visible jaundice in the first week after birth. Preterm neonates may have further decreased ability to conjugate and clear bilirubin [3]. The imbalance between production and clearance typically stabilizes by around 4 days after birth [4]. However, other factors manifesting in the newborn period can further affect bilirubin production and clearance, for example, isoimmune hemolytic jaundice from maternal blood type mismatch and transplacental transmission of maternal immunoglobulins or inadequate enteral intake resulting in dehydration, decreased bile clearance, and increased enterohepatic circulation.

### Bilirubin-Induced Morbidity

Although lower levels of hyperbilirubinemia are generally well tolerated by newborn infants, at sufficiently high concentrations, unconjugated bilirubin, presumably unbound to albumin, can cross the blood-brain barrier with potentially devastating consequences [5]. The manifestations of bilirubin-induced neurological dysfunction range from sleepiness, lethargy, discoordinated suck reflex, and high-pitched cry to abnormal muscle tone, athetosis, oculomotor paralysis, and opisthotonos, with associated sensorineural hearing loss and intellectual deficits. Extremely severe cases may result in seizures, coma, and death. Kernicterus originally referred to the pathologic finding of yellow bilirubin staining of the deep nuclei of the brain but is now also used to describe the syndrome of severe bilirubin encephalopathy.

### Phototherapy

Phototherapy is an effective treatment to prevent bilirubin-associated morbidity [6]. Absorption of light through the dermis and subcutaneous tissue induces photochemical changes in bilirubin to produce more hydrophilic isomers and derivatives that can be excreted in bile and urine without the need for conjugation. A visible spectrum of blue light from 460 nm to 490 nm in wavelength appears to have maximal efficacy in both penetrating tissue and formation of bilirubin photoproducts. Although phototherapy is not known to affect the rates of bilirubin production, effective administration is often able to increase bilirubin clearance to a rate greater than the rate of ongoing production, thereby lowering the total serum bilirubin concentration.

### Consensus Clinical Guidelines

Clinical guidelines have been developed to assist in the management of neonatal hyperbilirubinemia, including specifying thresholds at which phototherapy or other therapies should be provided [1]. The availability of effective treatments and the potentially devastating consequences of not initiating therapy have made it difficult to develop evidence-based guidelines, for example, via randomized controlled clinical trials or systematic observational studies. Therefore, currently available guidelines are largely consensus based.

There is not a single universally accepted guideline. An informal international survey conducted during the development of the Norwegian guidelines [7] for the treatment of neonatal jaundice reported that 18 of the 28 countries surveyed had national consensus treatment guidelines, including the United States [8], South Africa [9], Canada [10], Israel [11], the United Kingdom [12,13], and Norway [7]. They found that these guidelines differed considerably in the recommended total serum bilirubin level at which phototherapy should be initiated, indications for exchange transfusion, addressing the preterm population, use of transcutaneous bilirubinometry, when phototherapy should be discontinued, and recommended follow-up at or after discharge.

Of the identified national guidelines for the management of neonatal hyperbilirubinemia, 14 of 16 included recommendations for late preterm infants (typically born at 35 weeks of gestation or later) and 10 of 16 for early preterm infants. Although less in number, guidelines have also been developed specifically for the preterm population, again consensus based [14-16]. There are also unpublished locally developed treatment practices for preterm infants [17]. For example, in several of the Boston area teaching hospitals, an informal and unpublished rule of thumb for preterm infants is to divide the birth weight in grams by 200 as the phototherapy threshold in mg/dL (eg, 1500 g birth weight yielding a phototherapy threshold of 7.5 mg/dL) and twice that value as an exchange transfusion threshold.

### Rebound Hyperbilirubinemia

With the implementation of universal bilirubin screening of newborn infants during birth hospitalization, clinical practice guidelines advise whether to initiate phototherapy (although strict adherence to guidelines varies [18]), but less often provide direction on when to discontinue phototherapy and whether reinitiation of treatment may be required because of rebound hyperbilirubinemia.

Rebound bilirubin, in general, refers to an increase in the bilirubin level after discontinuation of phototherapy, likely related to the removal of the additional bilirubin clearance provided by phototherapy and the resultant return to net balance of greater bilirubin production than clearance. However, the specific definitions of rebound bilirubin vary considerably. Some definitions include the change in bilirubin level on the first follow-up serum bilirubin at any time up to 30 hours after discontinuation of phototherapy [19], between 4 hours and 48 hours after discontinuation [20], within 12 hours [21], or after approximately 6 hours [22]. Over time, the definition began to incorporate the concept of rebound to significant hyperbilirubinemia. The choice of significance could be an arbitrarily chosen constant threshold [23], a measurement between 18 hours and 30 hours after discontinuation that prompted reinstitution of phototherapy [24], or an increase at any time that resulted in exceeding the age-specific threshold of a specified clinical guideline to initiate phototherapy [25].

### Predictive Models

Defining rebound hyperbilirubinemia as exceeding the phototherapy initiation threshold of a practice guideline raises the possibility of developing predictive models to provide clinical decision support.

Predictive models can be generated by a class of statistical approaches referred to as supervised machine learning [26]. With supervised learning, a model is trained using a data set containing predictive features and their known target outcomes, with the aim that the trained model can later be used on a new set of the same predictive features to predict unknown outcomes. The goal might be a classification task—for example, predicting the likelihood of survival, readmission, or need to initiate phototherapy—or a regression task to calculate a continuous numeric outcome, such as a laboratory value. Some examples of machine learning models are as familiar as linear regression (ordinary least squares), which performs a regression prediction, and logistic regression, which performs a classification task despite its historic name. Different machine learning models differ in their approach and the flexibility with which they can predict outcomes. For example, both linear and logistic regression are in the family of generalized linear models and are relatively inflexible as a unit change in the value of each predictor produces a constant linear change in the output. More flexible models may be able to better fit the training data and perform better with new predictions but risk overfitting the training data, resulting in poorer performance on new, previously unseen data, that is, poor model generalization. Examples of strategies to limit overfitting include choosing less-flexible models or applying an approach called regularization that applies a penalty for larger model coefficients. Inappropriate use of too many predictors can also contribute to overfitting as high model flexibility can allow learning what is effectively noise and not signal in the predictive features of the training set. Owing to these risks, in general, it is best to evaluate a predictive model’s performance on data that was not previously used for training. Approaches to achieve this include using a completely separate training set and held-out test set or using K-fold cross-validation to partition the data and then training and evaluating models on each partition.

Chang et al [27,28] developed and subsequently simplified a logistic regression model to predict the need to resume phototherapy after an initial treatment episode with decision thresholds defined by the American Academy of Pediatrics (AAP) consensus treatment guidelines [8]. The choice of this specific guideline restricts its applicability to newborn infants born at ≥35 weeks of gestation. As clinical guidelines can vary significantly, the ability to generalize the published model to different guidelines may be limited. Another potential issue is assuming the validity of applying an age-specific treatment guideline, which was developed from a nomogram derived from a cohort of normal newborn infants without any previous phototherapy treatment, on infants who may have received varying duration and intensity of phototherapy. In these published models, there is no prediction distinction between a newborn infant who had phototherapy initiated very early because of the rapid development of jaundice (perhaps related to hemolysis) and another infant who had phototherapy initiated several days after birth as long as their bilirubin levels for a given age were subsequently the same after phototherapy. Moreover, the model can only be applied after an initial episode of phototherapy; it cannot be used to predict the need to initiate a first episode of phototherapy or account for multiple previous episodes of phototherapy.

### Aims of This Study

A more general approach that predicts actual bilirubin values, rather than exceeding thresholds defined within a particular treatment guideline, and not limited by gestational age or by restrictions on phototherapy utilization, might be helpful. By predicting actual bilirubin values, the approach could provide clinical decision support related to any given clinical guideline, including those developed for preterm infants. Training more flexible models than generalized linear models might improve prediction performance. This study aims to (1) develop and compare multiple predictive models of follow-up total serum bilirubin measurements that could be utilized regardless of gestational age or previous treatment with phototherapy; (2) to compare accuracy with clinician predictions; and (3) to demonstrate an example application to one specific clinical guideline.

## Methods

### Patient Cohort

The subjects of this retrospective study were newborn infants born at any gestation between June 2015 and June 2019 at 4 birthing hospitals in Massachusetts within the Partners HealthCare system. The hospitals provided a range of levels of neonatal care [29], with 2 hospitals providing up to level 2 care, 1 hospital providing up to level 3, and 1 hospital providing up to level 4. As the prediction target was a follow-up total serum bilirubin measurement obtained <72 hours after a previous measurement, the inclusion criteria were 2 bilirubin measurements <72 hours apart within the first 10 days after birth. There were no other exclusion criteria.

### Features of the Predictive Model

Data from inpatient encounters were abstracted from the electronic health record (EHR) by database query and included gestational age at birth, birth weight, gender, maternal age, gravida, para, race and ethnicity, route of delivery and whether the delivery was vacuum assisted or forceps assisted, 1-min and 5-min Apgar scores, maternal and baby blood type and Rh, baby direct Coombs, and initial baby hematocrit. Data from the first 10 days after birth included total serum bilirubin measurements, inpatient phototherapy start and stop times based on physician orders, weights, enteral feeds, urine output, and stools. Feature engineering included encoding nonnumeric (categorical) predictors to binary features of whether known to be present and included maternal race (White, Black, Hispanic, or Asian); ABO incompatibility as maternal blood type O and baby blood type A, B, or AB; Rh mismatch as maternal Rh negative and baby Rh positive; baby direct Coombs positive; cesarean delivery; forceps-assisted delivery; and vacuum-assisted delivery. If categorical data were unavailable, the feature was set to not known to be present. Median imputation was used for numeric features with missing data. Birth weight Z-score was calculated as described previously [30,31].

**Figure 1:**
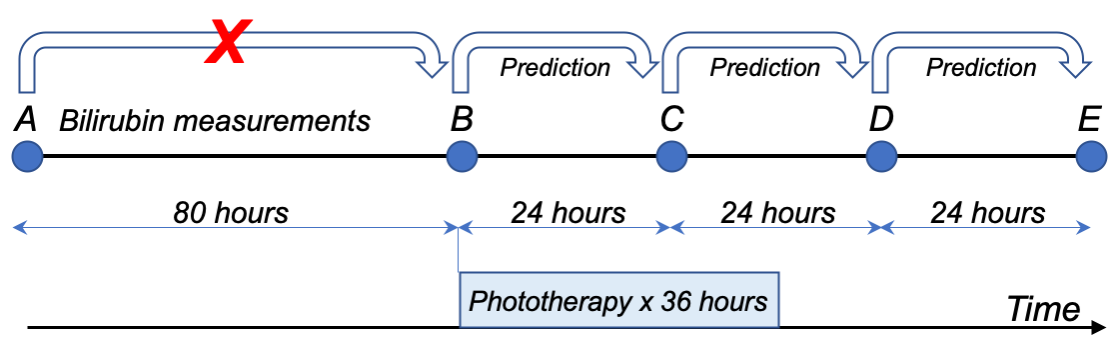
Schematic of a data inclusion mechanism for a hypothetical individual with 5 bilirubin measurements, A through E. The blue box represents the time period under phototherapy. Model training used features at the time of a bilirubin measurement to predict the value of a subsequent measurement ≤72 hours later. The predictive feature of fraction of time under phototherapy was 100% between B and C, 50% between C and D, and 0% between D and E. Data from bilirubin measurement A to predict B were not included for model training because the subsequent measurement was ≥72 hours later.

Individuals frequently had >2 bilirubin measurements, permitting multiple prediction targets. The goal of prediction was to only use information available at the time of a given bilirubin measurement (the *current* measurement) to predict the subsequent measurement. Available information included age in hours, current measurement, previous bilirubin rate of rise, and proportion of time under phototherapy between the previous and the current measurement. For the first bilirubin measurement, there would be no previous measurement; in this case, the time zero measurement was imputed as 2.0 mg/dL based on previous reports of umbilical cord bilirubin level and extrapolation from postnatal nomograms [4,32-35]. If 2 serum bilirubin measurements were recorded <2 hours apart, the earlier measurement was discarded as this generally reflected an erroneous first measurement. Additional features generated from the available data included fraction weight change from birth and counts of stools, urine output, and feeds on the previous calendar day. To make predictions, the only data permitted from after the current measurement were factors under clinician control, that is, number of hours until the target measurement and the fraction of that time that would be under phototherapy (between 0 for no phototherapy before the next measurement and 1 for continuous phototherapy until the next measurement). Figure 1 shows a schematic of the data inclusion mechanism and illustrates how the predictive feature of the fraction of time under phototherapy was calculated.

### Predictive Model Training

All data for patients born on or after February 1, 2019, were set aside as a held-out test set and not accessed before predictive model testing. The remaining data were used for model training.

Multiple supervised learning models were trained including linear models, linear models with interaction terms regularized via ridge regression or least absolute shrinkage and selection operator (LASSO), random forest, multilayer perceptron (a simple neural network with 2 densely connected hidden layers using the rectified linear unit nonlinear activation function), long short-term memory (LSTM) neural network, and Xgboost. Feature selection was explored by best subset selection for the linear model without interaction terms and variable importance for random forest and Xgboost. To improve neural network convergence, numeric predictors were centered and scaled by subtracting the mean and dividing by the SD of the predictors in the training set; both the validation and test sets were centered and scaled using the training set. The training set for the LSTM neural network was generated by creating a moving window of up to 4 time steps (zero-padded for the first 3 time points), allowing a memory of previous predictors. Analysis was performed using the R statistical programming language (R Core Team, 2018) [36]. Multimedia Appendix 1 includes the R code used for model training and references to the packages used. For data visualizations, smoothed conditional mean curves with 95% CIs were generated using the ggplot2 package [37].

### Comparison With Clinician Accuracy

From February 2019, a convenience sample of clinician predictions of follow-up bilirubin measurements was obtained by identifying currently admitted newborn infants at 1 hospital who had a recent bilirubin measurement and a provider clinical order for a follow-up bilirubin level to be obtained within the next 72 hours. Clinicians actively providing care for that neonate were approached and asked to provide predictions. Participation was voluntary and no information identifying the clinician was recorded other than the role group. Role groups included attending board-certified neonatologists, advanced practitioners (including neonatal nurse practitioners, neonatal-perinatal medicine fellows, and pediatric hospitalists with primary roles in the newborn intensive care unit [NICU]), pediatric residents (either interns or seniors during their NICU rotation), and bedside nurses (neonatal nurses, all in the level 2 and level 3 nurseries). Clinicians were asked to use all available information, including data not documented in the EHR, for example, team discussions during bedside rounds, conversations with parents and lactation consultants, etc.

### Statistical Analysis

Comparisons were performed using the *t* test (either paired or unpaired), analysis of variance, Wilcoxon rank-sum, Kruskal-Wallis, or Pearson chi-square test, as appropriate. When multiple pairwise comparisons of paired *t* tests were performed, multiple testing adjustment was performed using the Holm method. The absolute value of prediction errors is nonnegative, which results in a right-skewed distribution; therefore, in general, medians and IQRs are reported below. However, in pairwise comparisons of models, the differences in absolute errors were distributed more normally (data not shown). The confidence interval for the area under the receiver operator characteristic (AUROC) curve was obtained using the method of Hanley [38].

### Human Subjects’ Research

This study was approved by the Partners Human Research Committee institutional review board.

## Results

### Patient Cohort Characteristics

A total of 52,149 babies born between June 2015 and June 2019 were identified, of whom 46,361 were born before February 1, 2019. The 5788 babies born after February 2019 were set aside as a held-out test set and not accessed until predictive model evaluation.

Of the patients born before February 2019, 9723 babies had at least 2 total serum bilirubin measurements <72 hours apart within the first 10 days after birth and were included in the training set, whereas the remaining 36,638 babies were excluded, as detailed in Multimedia Appendix 2. The patients included in the training set tended to be of lower gestational age, lower birth weight, lower birth weight Z-score, and lower 1-min and 5-min Apgar scores; male; C-sectioned; forceps assisted; vacuum assisted; ABO-mismatched; absence of Rh mismatch; baby direct Coombs positive; and of maternal race Asian, Black, or not White. Of the patients in the training set, the median number of serum bilirubin measurements was 3 (IQR 2-5) and 34.34% (3339/9723) received phototherapy. There were significant missing data (>10%) in both the included and excluded patients for maternal and baby blood type and Rh, baby direct Coombs, and baby hematocrit. If the maternal blood type was O, the baby’s blood type was less likely to be missing (1017/18,930, 5.37%). Similarly, if the mother was Rh negative, the baby’s Rh status was unlikely to be missing (57/4919, 1.16%).

### Predictive Model Training

Of the 9723 babies in the training set, there were a total of 37,151 total serum bilirubin measurements resulting in 27,428 training examples. After feature engineering, 34 candidate predictors were available for model training, including 22 that did not vary with time (gestational age at birth; birth weight; birth weight Z-score; gender; 1-min and 5-min Apgar scores; cesarean versus vaginal delivery; forceps assistance; vacuum assistance; maternal age; gravida; para; maternal race Asian, Black, Hispanic, or White; ABO blood type mismatch; Rh mismatch; baby direct Coombs status; baby initial hematocrit; age; and value of first total serum bilirubin measurement) and 12 predictors that varied with time (current age; current bilirubin level; fractional weight change; count of breast milk, formula and donor human milk feeds, urine output and stools; last rate of rise; last proportion of time under phototherapy; and time to next measurement and fraction of that time under phototherapy). For the linear models, the quadratic age-squared term was added to account for the nonlinearity of bilirubin trajectories with age [4].

During the initial model exploration, it quickly became apparent that a limited number of predictive features would be sufficient for near-optimal model performance. For the simple linear model, the best subset and stepwise forward feature selection chose the same features until the 13th predictive feature was added, but showed limited improvement after the seventh feature (minimal R^2^ statistic improvement from 0.783 to 0.785). The features selected, in order of importance, included current result, proportion phototherapy before target measurement, current age, previous proportion of phototherapy, current age squared, time to target measurement, count of breast milk feeds, and first bilirubin measurement.

Random forest and Xgboost models are able to report predictive feature importance contributing to model accuracy. Providing all 34 predictive features to the random forest and Xgboost models and inspection of the variable importance plots also suggested that a limited number of features would provide near-maximal predictive accuracy. For the Xgboost model, the top 8 features included current result, last rate of rise, proportion phototherapy, time to target measurement, birth weight, gestational age, first bilirubin measurement, and current age; each of the remaining 26 features contributed <1% to Xgboost variable importance (data not shown). For the random forest model, the top 8 features included time to target measurement, proportion phototherapy, previous rate of rise, current result, current age, birth weight Z-score, previous proportion of phototherapy, and count of formula feeds.

The 8 features selected for final predictive model training were current result, last rate of rise, proportion of time under phototherapy between the current and the future target measurement, time to target measurement, gestational age, current age, previous proportion of time under phototherapy, and fractional weight change from birth. All models used these features except the age-squared term that was included for the linear models (to allow for nonlinear response with age). The last rate of rise and previous proportion of time under phototherapy were excluded from the LSTM model as those features were available via the preceding time step. Seven predictive models and 1 negative control were generated with the training set (Textbox 1; further detailed in Multimedia Appendix 1).

**Predictive models and descriptions.**

- *current*: Negative control, predicting the current bilirubin level as the subsequent level
- *lm*: Linear model with no interaction terms; includes quadratic age-squared term
- *ridge*: Linear model with all combinations of predictors as interaction terms and ridge regression regularization (L2 norm) selected by 10-fold cross-validation for coefficient shrinkage
- *lasso*: Similar to *ridge* but using least absolute shrinkage and selection operator (LASSO) regularization (L1 norm) for coefficient shrinkage and implicit feature selection
- *nn*: Multilayer perceptron (a simple neural network) with 2 fully connected hidden layers
- *lstm*: Long short-term memory recurrent neural network with 4 time steps feeding into a single hidden layer
- *rf*: Decision tree-based random forest ensemble with 500 trees
- *xgboost*: Decision tree-based XGBoost ensemble model with 500 boosting iterations

### Predictive Model Assessment

Predictive model performance was assessed using the held-out test set. Of the 5788 babies born after February 2019, 1224 had at least 2 total serum bilirubin measurements <72 hours apart within the first 10 days after birth, with a total of 4544 total serum bilirubin measurements resulting in a test set of 3320 examples.

For each prediction, the error is defined by the predicted value minus the actual value, with positive and negative values reflecting predictions that are too high or too low, respectively. Prediction models often have an overall mean prediction error of 0; simplistically, if the prediction is equally likely to be too high (positive error) or too low (negative error), the mean error may be near 0. Therefore, to assess the model performance, the absolute value of the prediction errors, which can be considered the magnitude of the error, was calculated.

Table 1 summarizes the predictive performance of all 8 models, which included a negative control and 7 models trained by supervised learning. The second and third columns show the mean and median absolute value of prediction errors for the 3320 test set examples for each model. The Xgboost model had the lowest mean (1.04 mg/dL, SD 0.99) and median (0.78 mg/dL) absolute values of prediction error, that is, for the Xgboost model, 50% of the test set predictions were within 0.78 mg/dL of the actual value.

**Table 1:** Pairwise comparison of the predictive models.

Model^a^	MAE^b^ (SD)	Median (IQR)^c^	*P* value^d^						
			current	lm	ridge	lstm	lasso	nn	rf
current	2.105 (1.674)	1.800 (0.900-2.900)	N/A^e^						
lm	1.325 (1.208)	0.997 (0.476-1.808)	<.0001	N/A					
ridge	1.175 (1.095)	0.893 (0.420-1.609)	<.0001	<.0001	N/A				
lstm	1.121 (1.142)	0.809 (0.363-1.493)	<.0001	<.0001	.0056	N/A			
lasso	1.075 (1.036)	0.802 (0.365-1.456)	<.0001	<.0001	<.0001	.0067	N/A		
nn	1.053 (1.007)	0.791 (0.362-1.407)	<.0001	<.0001	<.0001	<.0001	.056	N/A	
rf	1.050 (1.003)	0.782 (0.355-1.438)	<.0001	<.0001	<.0001	<.0001	.090	.74	N/A
xgboost	1.038 (0.989)	0.776 (0.355-1.427)	<.0001	<.0001	<.0001	<.0001	.0045	.29	.29

^a^Models are as described in Textbox 1.

^b^MAE: mean absolute error of bilirubin level predictions with SD (mg/dL) on the held-out test set (n=3320).

^c^Median absolute error of bilirubin level predictions and IQR (mg/dL).

^d^*P* values for pairwise model comparisons by paired *t* test with Holm adjustment for multiple testing.

^e^N/A: not applicable.

To assess the performance of each model with respect to each other model, a total of 28 pairwise comparisons of the 8 models’ predictions on the same 3320 test set examples, including the negative control, were analyzed by using a paired *t* test with Holm adjustment for multiple testing (Table 1, right-most 7 columns). Xgboost performance (Table 1, last row) was statistically significantly better than the negative control, simple linear model, ridge regression, LSTM neural network, and LASSO models (*P* values from <.0001 to .0045), but was not statistically significantly superior to the simple neural network (*P*=.29) or random forest (*P*=.29) models.

**Figure 2:**
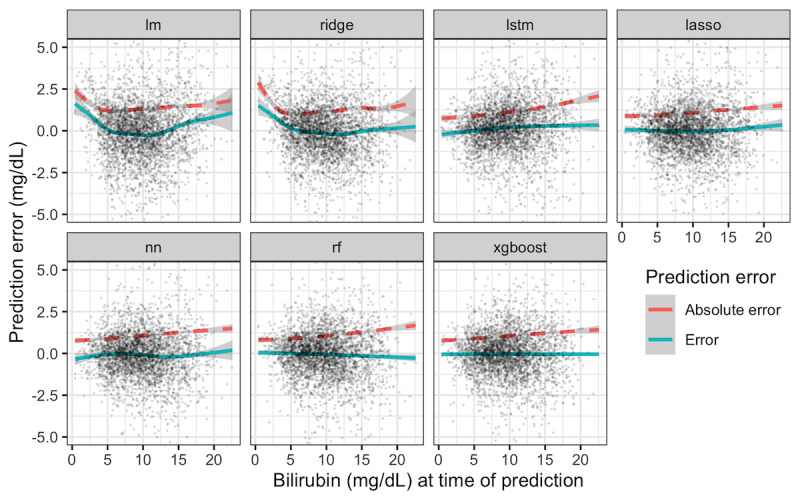
Model prediction errors versus bilirubin level at time of prediction. Each panel depicts the performance of a single predictive model, as described in Textbox 1. Each point represents the error of a single prediction in the test set (n=3320, over 98% visible within ±5 mg/dL error). The curves show the smoothed mean error (blue solid) and mean absolute value of error (red dashed); the gray band is the 95% CI of the mean.

Although Table 1 summarizes model performance across the entire test set, the greatest clinical concern is high bilirubin levels. To visualize whether performance was impacted by bilirubin level, prediction error was visualized with respect to the bilirubin value at the time of prediction for each of the models (Figure 2). Each point represents the error of a single prediction. For all models, the absolute value of the prediction error with respect to bilirubin level at the time of the prediction (Figure 2, red dashed lines) tended to increase at higher starting bilirubin levels, increasing from approximately 0.8 to 1.6 mg/dL as the starting bilirubin varied from 0 to 20 mg/dL for the neural network, random forest, and Xgboost models. However, the simple linear and ridge regression models also demonstrated a larger error magnitude at the low range of current bilirubin levels. The blue solid line represents the mean error versus the bilirubin level at the time of prediction. The Xgboost model demonstrates a mean prediction error of near 0 across all bilirubin values at the time of prediction (xgboost panel, blue line). In contrast, the simple linear and ridge regression models tend to predict values that are too high when bilirubin values are low at the time of prediction (blue line >0 at low starting bilirubin levels).

### Comparison With Clinician Accuracy

Model performance was also assessed by comparing predictions made by the models with prospective predictions made by clinicians participating in the clinical care of newborn infants. A convenience sample of 210 predictions made by clinicians at 1 hospital was compared with model predictions, all from the held-out test set. The clinicians included attending neonatologists, advanced practitioners (neonatal-perinatal medicine fellows, neonatal nurse practitioners, and pediatric hospitalists with primary responsibilities in the NICU), pediatric residents (interns and seniors), and bedside nurses (in the level 2 and level 3 nurseries). All predictive models other than the negative control had a lower absolute error than the clinician’s predictions (Table 2).

**Table 2:** Absolute errors of clinician and model predictions.

Model^a^	Mean (SD)^b^	Median (IQR)^c^	Clinician error difference^d^	*P* value
clinicians	1.38 (1.31)	1.10 (0.60-1.80)	N/A^e^	N/A
current	1.86 (1.55)	1.50 (0.80-2.58)	–0.49 (–0.68 to –0.29)	<.0001
lm	1.19 (1.03)	0.94 (0.50-1.67)	0.19 (0.04 to 0.34)	.0109
ridge	1.14 (1.01)	0.97 (0.48-1.47)	0.23 (0.10 to 0.36)	.0005
lstm	1.08 (1.01)	0.91 (0.37-1.55)	0.29 (0.14 to 0.44)	.0002
lasso	1.08 (0.95)	0.94 (0.51-1.38)	0.30 (0.17 to 0.43)	<.0001
nn	1.06 (1.02)	0.87 (0.34-1.36)	0.32 (0.18 to 0.45)	<.0001
rf	1.04 (0.91)	0.76 (0.34-1.48)	0.34 (0.20 to 0.48)	<.0001
xgboost	1.01 (0.90)	0.88 (0.37-1.41)	0.37 (0.22 to 0.52)	<.0001

^a^Models are as described in Textbox 1, with 210 predictions made by each model. Clinician predictions were from all role groups (attendings, advanced practitioners, residents, and nurses).

^b^Prediction mean absolute error and SD (mg/dL).

^c^Prediction median absolute error and IQR (mg/dL).

^d^Mean error differences (mg/dL, clinician absolute error minus model absolute error) with 95% confidence range and comparisons by paired *t* test. Positive values reflect higher prediction errors by clinicians.

^e^N/A: not applicable.

Clinician accuracy may differ by role (Table 3), but because predictions were made on different subsets of patients, accuracy by role group could not be directly compared with paired testing. Although advanced practitioners and attendings made predictions with lower mean absolute error (MAE), these predictions were made on measurements for which the simple neural network also had the lowest MAE, that is, this subset may have made it easier to make accurate predictions. When comparing predictions made by clinicians in each role with predictions made by the neural network, clinicians had statistically significant higher errors for all except the nursing group, which had the lowest number of predictions (n=31), potentially limiting statistical power.

**Table 3:** Clinician prediction accuracy by role and comparison with the neural network predictive model.

Role	Clinician MAE (SD)^a^	Clinician median error (IQR)^b^	Model MAE (SD)	Model median error (IQR)^b^	Mean error difference^c^	*P* value
All clinicians (n=210)	1.38 (1.31)	1.10 (0.60-1.80)	1.06 (1.02)	0.87 (0.34-1.36)	0.32 (0.18 to 0.45)	<.0001
Advanced practitioner (n=74)	1.17 (1.09)	0.90 (0.50-1.40)	0.93 (0.76)	0.83 (0.34-1.31)	0.24 (0.04 to 0.44)	.017
Attending (n=60)	1.36 (1.31)	1.20 (0.57-1.70)	0.99 (1.08)	0.76 (0.25-1.33)	0.37 (0.13 to 0.61)	.003
Resident (n=45)	1.54 (1.20)	1.10 (0.70-1.90)	1.12 (0.87)	1.09 (0.50-1.57)	0.43 (0.12 to 0.73)	.0071
Nurse (n=31)	1.65 (1.82)	1.20 (0.50-1.80)	1.40 (1.49)	1.07 (0.37-1.52)	0.25 (−0.25 to 0.75)	.32

^a^Clinician and neural network model prediction mean absolute error (MAE, mg/dL) and SD.

^b^Clinician and neural network model prediction median absolute error (mg/dL) and IQR.

^c^Mean clinician absolute error minus neural network absolute error (mg/dL), with 95% confidence range and comparisons by paired *t* test. Positive values reflect higher prediction errors by clinicians.

### Predicting Exceeding the Phototherapy Threshold

Although many treatment guidelines exist for the management of neonatal hyperbilirubinemia [7], the novelty of the approach described here is that the ability to predict actual bilirubin values allows the model to be adapted for different guidelines.

Two previously published models [27,28] predicted the need to resume phototherapy as recommended by consensus treatment guidelines [8], after an initial episode of phototherapy. The guidelines apply only to newborn infants at ≥35 completed weeks of gestation. Both models reported an AUROC curve of 0.88.

As a concrete example of adapting the general approach described in this study to a specific consensus-based guideline, the simple neural network was retrained to make a similar prediction—whether or not the next bilirubin measurement would exceed the phototherapy threshold—using the same 8 predictors described above by (1) limiting the data set to only those babies born at or after 35 weeks and (2) changing the prediction target to the dichotomous outcome exceeding the AAP-recommended phototherapy threshold [8]. The risk category was determined by gestational age and the presence of potential isoimmune hemolytic disease as reflected by a baby’s Coombs-positive result.

**Figure 3:**
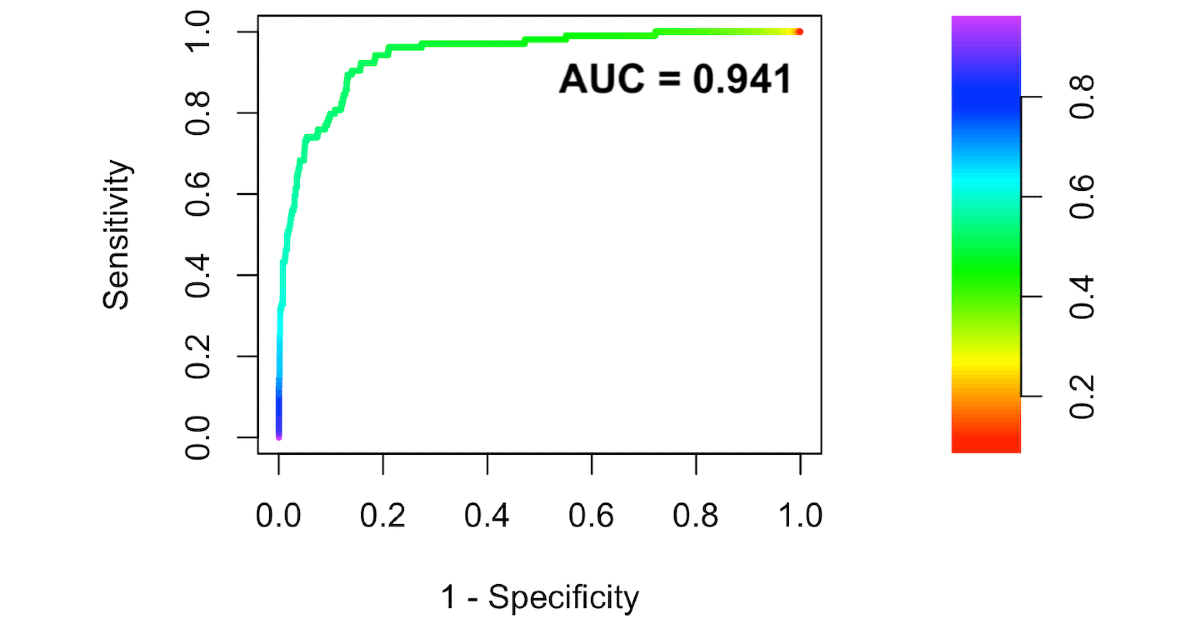
Receiver operator characteristic curve for neural network prediction of exceeding the American Academy of Pediatrics–recommended phototherapy initiation threshold on the subsequent bilirubin measurement in newborn infants ≥35 weeks of gestation. The area under the receiver operator characteristic curve was 0.941 (95% CI 0.910 to 0.973); a no-skill classifier would have an area under the receiver operator characteristic curve of 0.5. The color represents the decision threshold value corresponding to the sensitivity and specificity on the receiver operator characteristic curve. AUROC: area under the receiver operator characteristic curve; ROC: Receiver operator characteristic.

Limiting the data set to only those born after 35 weeks yielded a training set of 19,242 bilirubin prediction targets, of which 910 (910/19,242, 4.73%) exceeded the phototherapy threshold, and a held-out test set of 2449 prediction targets, of which 104 (104/2449, 4.25%) exceeded the phototherapy threshold. After training to make the binomial prediction, this neural network model performed well on the held-out test set with an AUROC curve of 0.941 (95% CI 0.910 to 0.973; Figure 3).

**Figure 4:**
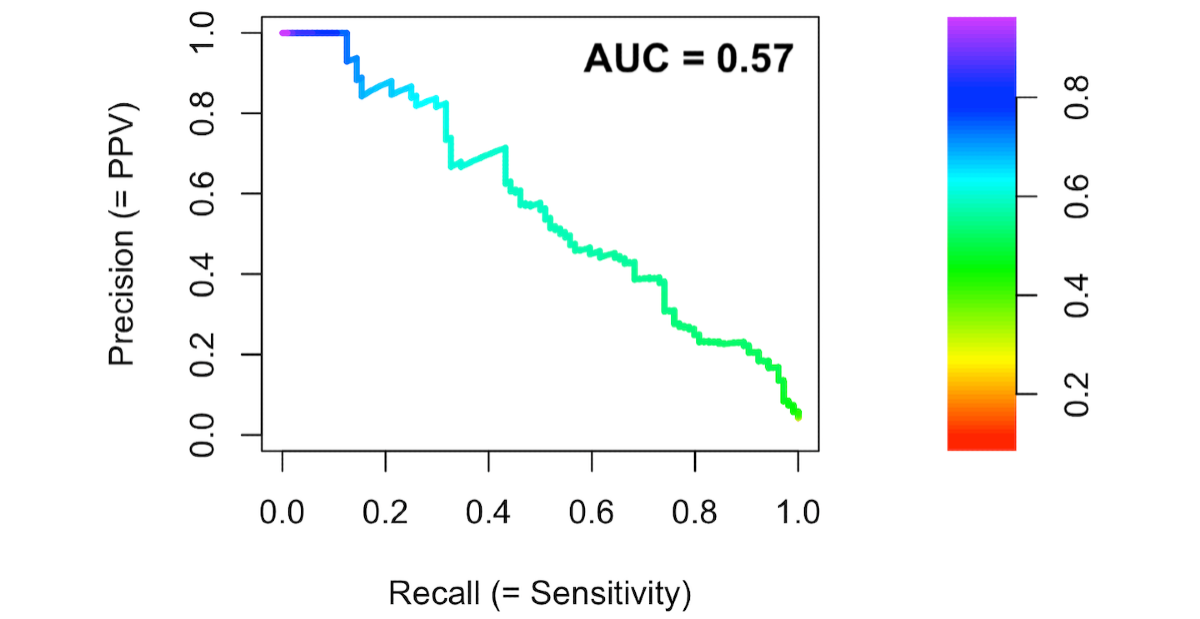
Precision recall curve for neural network prediction of exceeding the American Academy of Pediatrics–recommended phototherapy initiation threshold on the subsequent bilirubin measurement in newborn infants ≥35 weeks of gestation. The area under the precision recall curve was 0.573; for this data set, a no-skill classifier would have an area under the precision recall curve of 0.043. The color represents the decision threshold value corresponding to the precision and recall on the curve. AUPRC: area under the precision recall curve; PPV: positive predictive value.

The binary outcome in this data set is significantly imbalanced, with only 4.25% (104/2449) of the test set with a subsequent bilirubin measurement exceeding the phototherapy threshold. Although frequently used to report performance on binary classifiers, the AUROC curve can be misleading in imbalanced data sets for which the area under the precision recall (PR) curve (AUPRC) may be more informative [39,40]. The PR plot displays the relationship between precision (positive predictive value) and recall (sensitivity). Unlike the AUROC, for which a no-skill classifier would have an AUROC curve of 0.5, the no-skill baseline AUPRC varies depending on the class distribution. In this case, the baseline AUPRC for a no-skill classifier would be 0.0425. Figure 4 displays the PR curve performance of the neural network model, again with a good performance on the held-out test set with an AUPRC curve of 0.573.

## Discussion

### Principal Findings

Using a cohort of 10,947 babies from 4 hospitals born between 22 and 43 completed weeks of gestation and with 41,695 total serum bilirubin measurements, this study reports the generation and validation of machine learning models to predict follow-up bilirubin levels within 72 hours of a previous measurement during the first 10 days after birth that outperform clinician predictions. A set of 8 predictive features was sufficient for optimal model performance. This may be the first report of predicting specific bilirubin levels in newborn infants at any gestational age when taking into account the effect of phototherapy. This approach was also applied to predict subsequently exceeding the AAP-recommended phototherapy threshold for neonates born at ≥35 weeks of gestation, with good performance.

### Potential Applications

#### Prediction of Exceeding the Phototherapy Threshold of a Clinical Guideline

Previously published models [27,28] predicted the need to resume phototherapy as recommended by consensus treatment guidelines [8] after an initial episode of phototherapy and used logistic regression with only 2 or 3 predictors: gestational age, age at phototherapy initiation, and bilirubin level when phototherapy was discontinued relative to the phototherapy initiation threshold. These models achieved an AUROC curve of 0.88.

The models in this study differ in several ways: prediction of actual bilirubin values rather than a binary outcome, no restriction on gestational age, and taking into account previous episodes of phototherapy or phototherapy before the following measurement. This generalization allows application to any of the many clinical practice guidelines available for the management of neonatal hyperbilirubinemia.

In this study, the AAP 2004 guideline was chosen as a specific example of this approach. Retraining on a data set limited to babies born at ≥35 weeks of gestation and changing the prediction target to the binary outcome resulted in a neural network model with an AUROC curve of 0.941 (95% CI 0.910 to 0.973) on the held-out test set, which compares favorably with the previously reported logistic regression models (AUROC curve of 0.88). The improved performance might be related to taking into account the risk factor for isoimmune hemolytic disease (ie, the presence of baby’s Coombs-positive status) that alters the consensus guideline phototherapy initiation thresholds but is not directly included as a predictor in the previously published models.

Predicting subsequently requiring phototherapy when the baseline prevalence is only approximately 4% and with an imperfect predictive model, as demonstrated in the receiver operator characteristic and PR curves (Figures 3 and 4), is challenging and requires a tradeoff between sensitivity, specificity, and positive and negative predictive values.

The choice of a decision threshold depends greatly on the goal of the prediction. For example, if the goal is the relatively low-cost determination of which newborn infants should have a follow-up appointment with their pediatrician sooner rather than later after discharge, a lower decision threshold could be chosen that tolerates a higher false-positive rate. In the predictive model reported here, a decision threshold of 0.3 could be chosen, yielding a lower positive predictive value of 46%, but with a higher sensitivity of 58% and a negative predictive value of 98.1%.

In contrast, if the goal was to determine whether an infant’s discharge from the hospital should be delayed to initiate phototherapy, thereby increasing costs related to longer length of stay, a higher threshold might be chosen. For example, choosing a decision threshold of 0.6 for the model presented here would yield a positive predictive value that is increased to 87% but with sensitivity decreased to 25% and a negative predictive value of 96.8%. This choice would be to attempt to avoid prolonging the length of stay by keeping infants who are less likely to actually need phototherapy, but allowing a lower sensitivity and instead relying on outpatient follow-up to identify those infants who would need to be readmitted for phototherapy.

#### Application to Guidelines to Account for Previous Phototherapy

A more fundamental question is whether the AAP consensus treatment recommendations should be used after phototherapy has already been provided. This usage is not directly addressed or recommended in the 2004 guidelines [8] and there might be issues in the face validity of this practice. For example, early initiation of intensive phototherapy may effectively limit the initial increase in serum bilirubin but this may also result in a potentially falsely-reassuring low (subphototherapy threshold) age-specific bilirubin level, which may be followed by a resumed rapid rate of increase after discontinuation of phototherapy.

The predictive models reported in this study may be useful for developing treatment clinical decision support that predicts the risk of subsequently exceeding consensus-developed thresholds. The present treatment recommendations describe 3 phototherapy initiation curves for different risk categories that plateau at 15, 18, and 21 mg/dL for higher, medium, and lower risk, respectively. The models described here could be used to predict whether a chosen threshold might be exceeded in the future, when taking into account previous phototherapy as well as other clinical features (age, gestational age, empirically observed bilirubin rate of rise, etc).

#### Predictive Models for Neonatal Readmission

Readmissions of apparently healthy newborn infants are often associated with jaundice. In a retrospective study of 296,114 neonates discharged from 21 well-baby nurseries in the Intermountain Healthcare system, feeding problems (41%) and jaundice (35%) were frequently present in the 5308 early readmissions of apparently healthy neonates [2]. It is possible that the predictions made by the models reported here could be combined with other clinical features to develop a risk calculator for neonatal readmission. This risk assessment might identify higher-risk neonates for closer follow-up with primary care providers, visiting nurses, or lactation consultants. Previous unpublished work in assessing the risk of readmission of apparently healthy newborn infants discharged from a well-baby nursery used gestational age, age at time of discharge, weight loss, size for gestational age (eg, small for gestational age), maternal parity, and maternal race to yield a logistic regression predictive model with fair performance for predicting readmission (AUROC curve of 0.76; Joseph H Chou, unpublished work). An interesting future direction would be to determine whether the addition of present or predicted follow-up bilirubin measurements might improve performance. Ideally, this risk assessment would be performed automatically within the EHR, not requiring clinician input, and made available closer to the time of discharge.

#### Assessment of Adjunctive Treatment Efficacy

Intravenous immunoglobulin (IVIG) remains a recommended treatment modality for neonatal isoimmune hemolytic disease if total serum bilirubin continues to rise despite intensive phototherapy [8]. However, although some reports suggested a reduction in the need for exchange transfusion after IVIG administration, the practice remains controversial because most clinical trials were not blinded and a recent systematic meta-analysis suggested overall poor quality of evidence and an unknown benefit effect estimate [41]. In nonneonatal populations, IVIG has been rarely associated with worsening of hemolysis [42]; if this phenomenon is present in the neonatal population, there is the possibility of actually worsening jaundice from IVIG therapy.

In the models for bilirubin prediction reported in this study, administration of IVIG was not included as a predictive feature as it was a very rare occurrence (administered in 96 of the 52,149 [0.18%] babies in the starting population). In future work, it would be interesting to determine whether administration of IVIG affected the accuracy of predictions. For example, if IVIG administration was temporally associated with subsequent bilirubin predictions that were consistently too high, this could be interpreted as indirect evidence of IVIG resulting in a lower bilirubin rate of rise. Unlike previous unblinded studies that used avoidance of exchange transfusion as an outcome (albeit a clinically significant outcome), this proposed approach might be less susceptible to bias.

### Limitations

#### Implementation for Clinical Use

The models described in this study are not intended to be used directly by clinicians manually entering predictors, which would likely be too cumbersome for integration into care delivery workflows. Rather, the goal was to generate the best possible performing predictive models using only features easily accessible within the EHR for future integration into automated clinical decision support. Some EHR software providers are beginning to integrate analytics and artificial intelligence modules into their platforms, for example, the Cogito enterprise analytics module by Epic (Epic Systems Corporation) includes business intelligence and machine learning capabilities that is either embedded at the point of care or deployed via a cloud-based platform. A future goal would be to seamlessly provide advanced clinical decision support from within the EHR platform available during care delivery, without clinical provider intervention.

#### Data Quality and Completeness

The data were limited to those available from routine clinical care, thus predictions outside the norms of clinical care might be less accurate. However, the available data likely reflect the scenarios of highest interest to clinicians. Another concern is the potential for sampling bias. Follow-up bilirubin measurements may not have been initially planned but were instead obtained after visual recognition of unexpected jaundice, resulting in a bias toward higher bilirubin levels. A similar concern was raised in the AAP guidelines that the Bhutani nomograms should not be considered as describing the natural history of the neonatal bilirubin trend [4,8].

This study intentionally included all study subjects regardless of missing data on the basis that clinicians also often need to make decisions in the face of missing information. The goal of this study was not to generate predictions only if all desired information was available but rather to provide the best predictions possible using the available, potentially incomplete information. For model training, missing data were handled simplistically—median imputation and casting categorical predictors as whether or not known to be present. More sophisticated imputation techniques might yield better prediction performance.

The accuracy of the data extracted from the EHR was another concern. The duration and timing of phototherapy was determined by the timestamps of the clinician orders, which may not reflect actual start and stop times. It was not possible to differentiate between type or intensity of phototherapy or how frequently a baby was permitted to be removed from phototherapy. Of note, for the predictions provided by clinicians, providers actively providing care to the neonates were instructed to take all information into account, even if unavailable for predictive model training.

Some data were not available or were not extracted from the EHR and were therefore not available for model training. For example, glucose-6-phosphate dehydrogenase (G6PD) deficiency can result in jaundice secondary to hemolysis. However, G6PD status was not included as a predictive feature because the results from testing are typically not available in the neonatal time frame and the goal of this study was to generate models usable at the time of neonatal admission. As discussed earlier, IVIG therapy was not included in the model training because of its rarity (96/52,149, 0.18%). Exchange transfusion is another therapy for isoimmune hemolytic jaundice, sometimes utilized after failure of intensive phototherapy and IVIG administration. Exchange transfusion information was not readily extracted from the EHR, but its utilization is likely even less frequent than IVIG administration. As this information was not made available for model training, predictions are unlikely to be accurate in the setting of G6PD deficiency, IVIG administration, or exchange transfusion. However, although more accurate or more complete data for model training might improve prediction accuracy, it is notable that despite potential limitations to data quality, predictive model accuracy still surpassed that of clinicians.

#### Model Training

In this study, the data were explicitly split into a training set used for data exploration and model parameter choice and a held-out test set used for final model evaluation. A limitation of this approach is that each of the final models was trained once on the training set and evaluated once on the test set, limiting the ability to assess performance variance for each model.

An alternative approach would be to perform, for example, 10-fold cross-validation by combining the data into one large data set, creating 10 overlapping partitions of training and test sets and performing model training on each of the 10 partitions, each resulting in model evaluation on a different test set, allowing a better sense of model performance variance.

However, a major concern with this approach was the potential for data leakage and overestimation of performance. By the time clinician predictions were being collected, the previous data from June 2015 through February 2019 were explored for the initial steps of feature selection, hyperparameter choice (eg, regularization strength), and model architecture (eg, tree ensemble settings, multilayer perceptron structure). If the same data were used to evaluate performance via cross-validated model training, it may result in overly optimistic evaluation metrics.

Instead, the approach was to prevent any possibility of data leakage by completely separating out the post-February 2019 held-out test set and not accessing it until after the models were fully trained on the training set and then reporting model performance on the held-out test set. Another reason for this approach was the limited number of clinician predictions available, all after February 2019.

Future work could use newly acquired data to retrain the predictive models with the previously identified model parameters using K-fold cross-validation to allow a better estimate of model performance and variance.

#### Model Interpretability

Machine learning model interpretability is a significant issue [43-45]. The risk of trusting uninterpretable predictive models is the potential for failing to recognize when incorrect guidance is being provided. The models trained in this study range from those that are relatively interpretable (linear model with no interaction terms) to those whose functioning is obfuscated (neural network). As is typical in machine learning, a tradeoff between model simplicity and predictive performance was observed. Future work would aim to provide a means to understand model functioning when maintaining prediction accuracy. Another important direction for future work would be to provide confidence ranges for individual predictions.

#### Limitations of Laboratory Measurement

Nonsystematic laboratory variation of bilirubin analysis limits the achievable prediction accuracy. In a survey of instruments used for neonatal bilirubin measurement, the coefficient of variation (a measure of dispersion used to describe precision) ranged from 2% to 6% [46,47]. In this study, laboratory measurement precision was not evaluated; however, the test set median target bilirubin level was 10.6 mg/dL with a neural network model MAE of 1.05 mg/dL, suggesting that limitations in instrument precision (2% to 6% of 10.6 mg/dL is 0.21 mg/dL to 0.64 mg/dL) might account for a significant proportion of prediction error.

#### Transcutaneous Bilirubinometry

Transcutaneous bilirubin (TcB) measurement provides a convenient and noninvasive method for estimating serum bilirubin levels [48]. Nomograms have been developed for normal newborns born at ≥35 completed weeks of gestation [49-51], and a systematic review suggests that TcB measurement is reasonably accurate in the preterm population (born before 37 weeks of gestation) [52].

In this study, TcB data were not included as a predictive feature in model generation as they were noncontributory. In the 4 hospitals included in this study, the clinical practice was to routinely obtain a TcB measurement only in newborn infants born at or after 35 weeks of gestation. The TcB measurement was used mainly as a screening test; if concerning, serum bilirubin was immediately sent and all subsequent management was guided by serum bilirubin measurement.

As the goal of this study was to predict subsequent bilirubin measurements and to include infants born at <35 weeks of gestation and because any concerning TcB measurement was immediately followed by a serum bilirubin measurement, transcutaneous bilirubinometry, as utilized at the 4 hospitals in this study, did not provide additional information useful for model training. However, at other institutions with different practices, TcB measurement is likely to be useful for predictive modeling.

#### Generalizability

The prediction models were not externally validated to assess generalizability. However, it may be preferable for hospitals to train their own predictive models that account for local equipment and practices. For example, a hospital that routinely uses double overhead fluorescent tube banks of phototherapy as well as a bilirubin blanket under the baby will likely have different phototherapy efficacy compared with using only a single overhead fluorescent tube bank of phototherapy. The increasing accessibility of EHR data and relative ease of machine learning model training may make hospital-specific predictive models possible. Although personalized medicine has typically referred to practice tailored to individual patient variation, personalization should also be applied to hospital systems.

### Conclusions

Models were developed to predict follow-up total serum bilirubin levels in newborn infants <10 days old, which outperform clinicians. This may be the first report of models that predict actual bilirubin levels, are not limited to term and late preterm patients, and take into account the effect of phototherapy. The predictive features are readily accessible in EHRs, making integrated clinical decision support potentially feasible. Important directions for future work include improving model interpretability while maintaining prediction accuracy and providing confidence ranges of predictions.

## Appendix

Multimedia Appendix 1 R code for feature selection and model training.

Multimedia Appendix 2 Patient cohorts, excluded versus included in the training set.

## Abbreviations

AAP: American Academy of Pediatrics
AUPRC: area under the precision recall curve
AUROC: area under the receiver operator characteristic
G6PD: glucose-6-phosphate dehydrogenase
EHR: electronic health record
IVIG: intravenous immunoglobulin
LASSO: least absolute shrinkage and selection operator
LSTM: long short-term memory
MAE: mean absolute error
NICU: neonatal intensive care unit
PR: precision recall
TcB: transcutaneous bilirubinometry
