# Supplementary material for: Predictive Models for Neonatal Follow-Up Serum Bilirubin: Model Development and Validation
Source: JMIR Med Inform. 2020 Oct 29;8(10):e21222. doi: 10.2196/21222 (PMC7661258; doi:10.2196/21222)
Supplement: Multimedia Appendix 1 [file medinform_v8i10e21222_app1.doc]

## Supplementary Materials

## R code for feature selection and model training

To assist readers who might want to reproduce the methods of predictive model generation, the following code fragments are excerpted from the R code used to train the predictive models in this study and include the modifiable parameters and specific version numbers of the packages used at the time of model training. Links to the package source on the Comprehensive R Archive Network (CRAN) are also included. The %>% pipe operator is from the magrittr package (accessed via the tidyverse package). Model training was performed on a laptop computer running Mac OS X.

### Linear model feature selection

The choice of exhaustive best subset versus stepwise forward selection is determined by the method parameter (as exhaustive versus forward). nvmax specifies the maximum size of subsets to examine. The age-squared term is manually added to the lm call, as it was not present in the source dataframe.

library(leaps)
# leaps * 3.0 2017-01-10 [1] CRAN (R 3.5.0)
# https://cran.r-project.org/web/packages/leaps/index.html

lm_select <- regsubsets(next_result ~ . + I(age^2),
 data = df_train_full,
 method = 'exhaustive',
 nvmax = 30)

select_summary <- summary(lm_select)

select_summary

plot(select_summary$rsq) # monotonic improvement in R^2 statistic

### Simple linear model without interaction terms (lm)

The lm linear model function is part of the base R installation in the stats package. The age-squared term is manually added to the lm call, as it was not present in the source dataframe.

# R version 3.5.1 (2018-07-02)
# https://stat.ethz.ch/R-manual/R-devel/library/stats/html/lm.html

lmmodel <- lm(next_result ~ age + I(age^2) + prop_photo +
 result + time_to_next + wtchange + ga +
 last_prop_photo + last_rate,
 data = df_train_full)

pred <- predict(lmmodel, df_test)

### Linear model with interaction terms, with ridge and LASSO regularization (ridge, lasso)

By separating all of the predictors with * rather than +, all possible interaction terms are included. With all possible product combinations of 9 predictors (8 plus the age-squared term), a total of 2^9^ = 512 coefficients are included in the linear model, including the intercept term. To reduce overfitting, regularization was applied. Regularization via ridge regression versus LASSO is specified by the alpha parameter and is the only code difference between model training for the two. Regularization strength lambda is determined by 10-fold cross-validation in cv.glmnet (the default for nfolds is 10), and is chosen as the largest value of lambda such that training error is within 1 standard error of the minimum.

library(glmnet)
# glmnet * 2.0-18 2019-05-20 [1] CRAN (R 3.5.2)
# https://cran.r-project.org/web/packages/glmnet/index.html

x <- model.matrix(next_result ~ age * I(age^2) * prop_photo *
 result * time_to_next * wtchange * ga *
 last_prop_photo * last_rate,
 df_train_full)[, -1]

y <- df_train_full$next_result

# alpha = 0 for ridge regression; alpha = 1 for LASSO
cvfit <- cv.glmnet(x, y, alpha = 0)

pred <- predict(
 cvfit,
 newx = model.matrix(next_result ~ age * I(age^2) * prop_photo *
 result * time_to_next * wtchange * ga *
 last_prop_photo * last_rate,
 df_test)[, -1],
 s = "lambda.1se")

### Random Forest (rf)

The random forest training as performed below resulted in overfitting, as indicated by significantly inferior performance on the test set (MAE 1.05 mg/dL) compared to the training set (MAE 0.51 mg/dL), suggesting that the predictive model could be simplified. Random forest training, however, was relatively slow compared to simple neural network and Xgboost model training, and it did not appear that random forest was likely to outperform the other models, so optimization was not pursued.

library(randomForest)
# randomForest * 4.6-14 2018-03-25 [1] CRAN (R 3.5.0)
# https://cran.r-project.org/web/packages/randomForest/index.html

df_train_full <- data_train_full %>%
 select(next_result, age, prop_photo,
 result, time_to_next, wtchange, ga,
 last_prop_photo, last_rate)

df_test <- data_test %>%
 select(next_result, age, prop_photo,
 result, time_to_next, wtchange, ga,
 last_prop_photo, last_rate)

rfmodel <- randomForest(next_result ~ .,
 data = df_train_full,
 importance = TRUE,
 ntree = 500)

pred <- predict(rfmodel, df_test)

### Simple neural network (nn)

A multilayer perceptron (densely connected) with two hidden layers using the rectified linear unit (ReLU) nonlinear activation function, referred to as the simple neural network. Data was partitioned first into pre- and post-February 2019 data. The pre-February 2019 data was further partitioned randomly into training (80%) and validation (20%) datasets. To prevent information leakage, any given individual from before February 2019 who had multiple bilirubin training examples was only assigned into one of the two partitions. Input data was centered and scaled on the training data to speed convergence during training; both validation and test sets were also centered and scaled using the data from the training data, to prevent information leakage.

library(keras)
# keras * 2.2.4.1 2019-04-05 [1] CRAN (R 3.5.2)
# https://cran.r-project.org/web/packages/keras/index.html

model <- keras_model_sequential()

model %>%
 layer_dense(units = 40, activation = 'relu',
 input_shape = dim(X_train)[2]) %>%
 layer_dense(units = 10, activation = 'relu') %>%
 layer_dense(units = 1)

model %>%
 compile(
 loss = 'mean_absolute_error',
 optimizer = 'adam',
 metrics = list("mean_squared_error")
 )

history <- model %>%
 fit(
 x = X_train,
 y = y_train,
 validation_data = list(X_validate, y_validate),
 batch_size = 128,
 epochs = 30
 )

pred <- predict(model, X_test)[,1]

### Long short-term memory neural network (lstm)

Not included is code for preparation of the training data into tensors of the proper dimensionality. Window size (for number of time steps) in this study was 4, and when fewer than 4 time steps were available (e.g., for the first 3 bilirubin targets), the data was zero-padded on the left. For the pre-February 2019 data, the same training (80%) and validation (20%) partitions were used as for the simple neural network. Input data was centered and scaled on the training data to speed convergence during training; both validation and test sets were also centered and scaled using the data from the training data, to prevent information leakage.

library(keras)
# keras * 2.2.4.1 2019-04-05 [1] CRAN (R 3.5.2)
# https://cran.r-project.org/web/packages/keras/index.html

model <- keras_model_sequential()

model %>%
 layer_lstm(
 units = 10, # dimensionality of the output space
 batch_input_shape = c(1, window_size, dim(X_train)[3])
 ) %>%
 layer_dense(units = 5) %>%
 layer_dense(units = 1)

model %>%
 compile(
 loss = 'mean_absolute_error',
 optimizer = 'adam',
 metrics = list("mean_squared_error")
 )

history <- model %>%
 fit(
 x = X_train,
 y = y_train,
 validation_data = list(X_validate, y_validate),
 batch_size = 1,
 epochs = 10
 )

pred <- predict(model, X_test)[,1]

### Xgboost (xgboost)

For the pre-February 2019 data, the same training (80%) and validation (20%) partitions were used as for the simple neural network. However, the data did not need to be centered and scaled for Xgboost training efficiency.

library(xgboost)
# xgboost * 0.90.0.2 2019-08-01 [1] CRAN (R 3.5.2)
# https://cran.r-project.org/web/packages/xgboost/index.html

dtrain <- xgb.DMatrix(data = X_train, label = y_train)
dvalidate <- xgb.DMatrix(data = X_validate, label = y_validate)
watchlist <- list(train = dtrain, validate = dvalidate)

bst <- xgb.train(
 data = dtrain,
 max_depth = 6,
 eta = 0.2,
 gamma = 20,
 nrounds = 500,
 watchlist = watchlist,
 eval_metric = 'mae',
 verbose = 0
)

pred <- predict(bst, X_test)

### Simple neural network for binary outcome prediction

A nearly identically structured neural network was trained to predict the dichotomous outcome of exceeding the AAP-recommended phototherapy threshold. For the pre-February 2019 data, the same training (80%) and validation (20%) partitions were used as for the simple neural network but were subsequently filtered to gestation at 35 weeks or later. Input data was centered and scaled on the training data to speed convergence during training; both validation and test sets were also centered and scaled using the data from the training data, to prevent information leakage.

The only changes for binary outcome prediction from the previous simple neural network are:

- dataset limited to only babies at or greater than 35 weeks gestation (the population for which the AAP guidelines apply), resulting in a training set (n=15,324), validation set (n=3,918), and held-out test set (n=2,449)
- prediction target is binary (0 or 1), instead of a continuous numeric bilirubin level
- loss function is binary cross-entropy, instead of mean absolute error
- metric followed is accuracy, instead of mean squared error
- convergence is slower, so 100 training epochs were used instead of 30

library(keras)
# keras * 2.2.4.1 2019-04-05 [1] CRAN (R 3.5.2)
# https://cran.r-project.org/web/packages/keras/index.html

model <- keras_model_sequential()

model %>%
 layer_dense(units = 40, activation = 'relu',
 input_shape = dim(X_train)[2]) %>%
 layer_dense(units = 10, activation = 'relu') %>%
 layer_dense(units = 1)

model %>%
 compile(
 loss = 'binary_crossentropy', # changed from nn
 optimizer = 'adam',
 metrics = list("accuracy") # changed from nn
 )

# y_train, y_validate, and y_test changed to 0 / 1 outcome encoding
history <- model %>%
 fit(
 x = X_train,
 y = y_train,
 validation_data = list(X_validate, y_validate),
 batch_size = 128,
 epochs = 100 # changed from nn
 )

pred <- predict(model, X_test)[,1]
