# Supplementary material for: Predictive Models for Neonatal Follow-Up Serum Bilirubin: Model Development and Validation
Source: JMIR Med Inform. 2020 Oct 29;8(10):e21222. doi: 10.2196/21222 (PMC7661258; doi:10.2196/21222)
Supplement: Multimedia Appendix 2 [file medinform_v8i10e21222_app2.docx]

## Multimedia Appendix 2

## Patient cohorts, excluded versus included in the training set.

| Patient cohorts | Excluded (n=36,638) | Included (n=9723) | *P* value |
| --- | --- | --- | --- |
| Birthweight (grams) | 3374 (3060-3680) | 3060 (2455-3490) | <.001**^a^** |
| Birthweight Z-score | −0.025 (−0.034 to −0.016) | −0.102 (−0.120 to –0.083) | <.001**^b^** |
| Birth gestation (weeks) | 39.43 (38.71-40.29) | 38.29 (36.00-39.57) | <.001**^a^** |

| **Birth gestation category (weeks), n (%)** |
| --- |

|  | 22-25 | 24 (0.1) | 87 (0.9) | <.001**^c^** |
| --- | --- | --- | --- | --- |
|  | 26-28 | 9 (0.0) | 189 (1.9) | <.001**^c^** |
|  | 29-31 | 17 (0.0) | 366 (3.8) | <.001**^c^** |
|  | 32-34 | 92 (0.3) | 1163 (12.0) | <.001**^c^** |
|  | 35-37 | 4802 (13.1) | 2613 (26.9) | <.001**^c^** |
|  | 38-40 | 27,819 (75.9) | 4778 (49.1) | <.001**^c^** |
|  | 41-43 | 3875 (10.6) | 527 (5.4) | <.001**^c^** |

| **1-min Apgar** |
| --- |

|  | Median (IQR) | 8 (8-9) | 8 (7-8) | <.001**^a^** |
| --- | --- | --- | --- | --- |
|  | Missing, n (%) | 33 (0.09) | 25 (0.3) | <.001**^a^** |

| **5-min Apgar** |
| --- |

|  | Median (IQR) | 9 (9-9) | 9 (8-9) | <.001**^a^** |
| --- | --- | --- | --- | --- |
|  | Missing, n (%) | 33 (0.09) | 26 (0.3) | <.001**^a^** |
| Male gender, n (%) | | 18,442 (50.3) | 5201 (53.5) | <.001**^c^** |

| **Maternal age (years)** |
| --- |

|  | Mean (SD) | 32.80 (4.96) | 32.91 (5.41) | .044**^b^** |
| --- | --- | --- | --- | --- |
|  | Missing, n (%) | 100 (0.3) | 442 (4.5) | .044**^b^** |

| **Grava** |
| --- |

|  | Median (IQR) | 2 (1-3) | 2 (1-3) | <.001**^a^** |
| --- | --- | --- | --- | --- |
|  | Missing, n (%) | 100 (0.3) | 442 (4.5) | <.001**^a^** |

| **Para** |
| --- |

|  | Median (IQR) | 2 (1-2) | 1 (1-2) | <.001**^a^** |
| --- | --- | --- | --- | --- |
|  | Missing, n (%) | 100 (0.3) | 442 (4.5) | <.001**^a^** |

| **Cesarean delivery, n (%)** |
| --- |

|  | No | 26,081 (71.2) | 5095 (52.4) | <.001**^c^** |
| --- | --- | --- | --- | --- |
|  | Yes | 10,457 (28.5) | 4186 (43.1) | <.001**^c^** |
|  | Missing | 100 (0.3) | 442 (4.5) | <.001**^c^** |

| **Forceps assisted, n (%)** |
| --- |

|  | No | 36,098 (98.5) | 9132 (93.9) | <.001**^c^** |
| --- | --- | --- | --- | --- |
|  | Yes | 440 (1.2) | 149 (1.5) | <.001**^c^** |
|  | Missing | 100 (0.3) | 442 (4.5) | <.001**^c^** |

| **Vacuum assisted, n (%)** |
| --- |

|  | No | 35,011 (95.6) | 8774 (90.2) | <.001**^c^** |
| --- | --- | --- | --- | --- |
|  | Yes | 1527 (4.2) | 507 (5.2) | <.001**^c^** |
|  | Missing | 100 (0.3) | 442 (4.5) | <.001**^c^** |

| **Maternal blood type, n (%)** |
| --- |

|  | A | 11,411 (31.1) | 2534 (26.1) | <.001**^c^** |
| --- | --- | --- | --- | --- |
|  | AB | 1437 (3.9) | 353 (3.6) | <.001**^c^** |
|  | B | 4905 (13.4) | 1241 (12.8) | <.001**^c^** |
|  | O | 14,690 (40.1) | 4240 (43.6) | <.001**^c^** |
|  | Missing | 4195 (11.4) | 1355 (13.9) | <.001**^c^** |

| **Maternal Rh, n (%)** |
| --- |

|  | Negative | 3946 (10.8) | 973 (10.0) | <.001**^c^** |
| --- | --- | --- | --- | --- |
|  | Positive | 28,505 (77.8) | 7395 (76.1) | <.001**^c^** |
|  | Missing | 4187 (11.4) | 1355 (13.9) | <.001**^c^** |

| **Baby blood type, n (%)** |
| --- |

|  | A | 5202 (14.2) | 2365 (24.3) | <.001**^c^** |
| --- | --- | --- | --- | --- |
|  | AB | 312 (0.9) | 196 (2.0) | <.001**^c^** |
|  | B | 2006 (5.5) | 1139 (11.7) | <.001**^c^** |
|  | O | 12,229 (33.4) | 3412 (35.1) | <.001**^c^** |
|  | Missing | 16,889 (46.1) | 2611 (26.9) | <.001**^c^** |

| **Baby Rh, n (%)** |
| --- |

|  | Negative | 2728 (7.4) | 868 (8.9) | <.001**^c^** |
| --- | --- | --- | --- | --- |
|  | Positive | 17,146 (46.8) | 6307 (64.9) | <.001**^c^** |
|  | Missing | 16,764 (45.8) | 2548 (26.2) | <.001**^c^** |

| **ABO mismatch, n (%)** |
| --- |

|  | No | 29,004 (79.2) | 7024 (72.2) | <.001**^c^** |
| --- | --- | --- | --- | --- |
|  | Yes | 3951 (10.8) | 1729 (17.8) | <.001**^c^** |
|  | Missing | 3683 (10.1) | 970 (10.0) | <.001**^c^** |

| **Rh mismatch, n (%)** |
| --- |

|  | No | 30,129 (82.2) | 7834 (80.6) | <.001**^c^** |
| --- | --- | --- | --- | --- |
|  | Yes | 2537 (6.9) | 638 (6.6) | <.001**^c^** |
|  | Missing | 3972 (10.8) | 1251 (12.9) | <.001**^c^** |

| **Baby Coombs positive, n (%)** |
| --- |

|  | No | 15,568 (42.5) | 5522 (56.8) | <.001**^c^** |
| --- | --- | --- | --- | --- |
|  | Yes | 839 (2.3) | 909 (9.3) | <.001**^c^** |
|  | Missing | 20,231 (55.2) | 3292 (33.9) | <.001**^c^** |

| **Maternal Race** |
| --- |

| Asian, n (%) | 3454 (9.4) | 1245 (12.8) | <.001**^c^** |
| --- | --- | --- | --- |
| Black, n (%) | 3008 (8.2) | 1225 (12.6) | <.001**^c^** |
| Hispanic, n (%) | 1232 (3.4) | 359 (3.7) | .112**^c^** |
| White, n (%) | 22,053 (60.2) | 4691 (48.2) | <.001**^c^** |

| **Baby hematocrit** |
| --- |

|  | Mean (CI) | 50.10 (49.90-50.31) | 49.22 (49.02-49.42) | <.001**^b^** |
| --- | --- | --- | --- | --- |
|  | Missing, n (%) | 33,009 (90.1) | 4634 (47.7) | <.001**^b^** |
| # bili^d^ before 10 days (IQR) | | 0 (0-0) | 3 (2-5) | <.001**^a^** |
| Any phototherapy, n (%) | | 20 (0.1) | 3339 (34.3) | <.001**^c^** |

^a^Kruskal-Wallis rank-sum test.

^b^Linear model analysis of variance.

^c^Pearson chi-square test.

^d^Total serum bilirubin measurements.
